# Complete genome sequence of *Desulfocapsa sulfexigens*, a marine deltaproteobacterium specialized in disproportionating inorganic sulfur compounds

**DOI:** 10.4056/sigs.3777412

**Published:** 2013-04-15

**Authors:** Kai Waldemar Finster, Kasper Urup Kjeldsen, Michael Kube, Richard Reinhardt, Marc Mussmann, Rudolf Amann, Lars Schreiber

**Affiliations:** 1 Bioscience-Microbiology Section, Aarhus University, Ny Munkegade 116, Dk-8000 Aarhus C, Denmark; 2 Center for Geomicrobiology, Bioscience, Ny Munkegade 116, Dk-8000 Aarhus C, Denmark; 3 MPI *-*Molecular Genetics. Ihnestrasse 63-73. D-14195 Berlin-Dahlem. Germany; 4 MPI -Marine Microbiology, Celsiusstrasse 1, D-28359 Bremen, Germany

**Keywords:** Sulfur-cycle, thiosulfate, sulfite, sulfur disproportionation, marine, sediment

## Abstract

*Desulfocapsa sulfexigens* SB164P1 (DSM 10523) belongs to the deltaproteobacterial family *Desulfobulbaceae* and is one of two validly described members of its genus. This strain was selected for genome sequencing, because it is the first marine bacterium reported to thrive on the disproportionation of elemental sulfur, a process with a unresolved enzymatic pathway in which elemental sulfur serves both as electron donor and electron acceptor. Furthermore, in contrast to its phylogenetically closest relatives, which are dissimilatory sulfate-reducers, *D. sulfexigens* is unable to grow by sulfate reduction and appears metabolically specialized in growing by disproportionating elemental sulfur, sulfite or thiosulfate with CO_2_ as the sole carbon source. The genome of *D. sulfexigens* contains the set of genes that is required for nitrogen fixation. In an acetylene assay it could be shown that the strain reduces acetylene to ethylene, which is indicative for N-fixation. The circular chromosome of *D. sulfexigens* SB164P1 comprises 3,986,761 bp and harbors 3,551 protein-coding genes of which 78% have a predicted function based on auto-annotation. The chromosome furthermore encodes 46 tRNA genes and 3 rRNA operons.

## Introduction

The disproportionation of inorganic sulfur is a microbially catalyzed chemolithotrophic process, in which elemental sulfur, thiosulfate and sulfite serve as both electron donor and acceptor, and are converted to hydrogen sulfide and sulfate. Thus, the overall process is comparable to the fermentation of organic compounds and is consequently often described as “inorganic fermentation”. Disproportionation of thiosulfate and sulfite represent exergonic processes with ΔG^0’^ of -21.9 and -58.9 kJ mol^-1^ of substrate, respectively [1]. In contrast, the disproportionation of elemental sulfur is endergonic under standard conditions (ΔG^0’^ = 10.2 kJ mol^-1^ S^0^). However, the energy output depends on the concentration of hydrogen sulfide, and under environmental conditions, where concentrations of free hydrogen sulfide are low due to precipitation with iron and/or rapid oxidation, the process becomes exergonic - *e.g.* ΔG^0’^ = -30 kJ mol^-1^ S^0^ at a hydrogen sulfide concentration of 10^-7^ M and a sulfate concentration of 2.8 x 10^-2^ M [2,3]. Isotope tracer studies have shown that inorganic sulfur disproportionation is of environmental significance in marine sediments [4,5]. Furthermore it seems to be a very ancient mode of microbial energy metabolism that has presumably left significant isotopic signatures in the geological sulfur rock record [6,7].

The ability to disproportionate inorganic sulfur compounds has recently been documented for a number of anaerobic sulfate-reducing *Deltaproteobacteria*, in particular for species of the genera *Desulfocapsa*, *Desulfobulbus*, *Desulfovibrio* and *Desulfofustis* (see [8] for a review). Additionally, Milucka et al. [9] found first evidence for this process to occur among *Desulfobacteraceae* in association with methane-oxidizing *Archaea*. The authors proposed that the associated bacteria disproportionate sulfur that stems from sulfate reduction by the methanotrophic archaea and that is released in the form of disulfide.

The reaction pathways underlying thiosulfate and sulfite disproportionation have been partly resolved owing to studies of enzymatic activities in cell extracts [10,11]. However, the mechanism by which elemental sulfur is first accessed by the cell and later processed is enigmatic, and the genetic basis of the deltaproteobacterial disproportionation pathways are currently unclear.

The two validly described members of the deltaproteobacterial genus *Desulfocapsa*, *D. sulfexigens* SB164P12 [2] and *D. thiozymogenes* Bra2 [12] are both able to grow by disproportionating elemental sulfur, thiosulfate or sulfite under anaerobic conditions using CO_2_ as their sole carbon source. Unlike *D. thiozymogenes* and most members of their sister genera within the family *Desulfobulbaceae*, *D. sulfexigens* is unable to grow by sulfate reduction. This specialized energy metabolism qualifies *D. sulfexigens* as a relevant candidate model organism for studying the physiologically interesting and biogeochemically relevant process of disproportionation of inorganic sulfur compounds. Here we present a summary of the taxonomic classification and key phenotypic features of *D. sulfexigens* SB164P1 together with the description of its complete and annotated genome sequence.

## Classification and features

*Desulfocapsa sulfexigens* (sul.f.ex′i.gens. L. n.*sulfurum*, sulfur; L. v.*exigo*, to demand; M. L. part. adj. *sulfexigens*, demanding sulfur for growth) SB164P1^T^, DSM 10523^T^ [13] was isolated from a tidal flat in the bay of Arcachon at the southwest coast of France. It is a strictly meso- and neutrophilic anaerobic bacterium with rod-shaped cells that are motile by a polar flagellum (Table1). In addition to growing by disproportionating sulfite, thiosulfate and elemental sulfur, *D. sulfexigens* SB164P1^T^ also grows by reducing elemental sulfur with H_2_ as the electron donor, a process, which occurs concomitantly with elemental sulfur disproportionation in the presence of H_2_ (K. Finster unpublished results). When growing by elemental sulfur disproportionation in the presence of excess ferric iron as sulfide scavenger, pyrite and sulfate are the main end products of its dissimilatory metabolism. *D. sulfexigens* SB164P1^T^ grows autotrophically on bicarbonate, as ^13^C-bicarbonate is incorporated into cell material and biomass production is not stimulated by the presence of acetate in the growth medium [10]. The strain is routinely grown with ammonia as a nitrogen source but can also fix N_2_ (Unpublished data).

**Table 1:** Classification and general features of *D. sulfexigens* SB164P1 according to the MIGS recommendations [14]

MIGS ID	Property	Term	Evidence code
	Current classification	Domain *Bacteria*	TAS [15]
		Phylum *Proteobacteria*	TAS [16]
		Class *Deltaproteobacteria*	TAS [17,18]
		Order *Desulfobacterales*	TAS [18,19]
		Family *Desulfobulbaceae*	TAS [20,21]
		Genus *Desulfocapsa*	TAS [12,22]
		Species *Desulfocapsa sulfexigens*	TAS [2,23]
	Gram stain	negative	TAS [2]
	Cell shape	rod-shaped	TAS [2]
	Motility	motile	TAS [2]
	Sporulation	non-sporulating	TAS [2]
	Temperature range	mesophilic; optimum 30^0^ C	TAS [2]
	pH range	6.0 to 8.2	TAS [2]
MIGS-6.3	Salinity range	0.17 – 0.33 M Na^+^	TAS [2]
MIGS-22	Oxygen requirements	anaerobic	TAS [2]
	Carbon source	HCO_3_^-^	TAS [2]
	Energy source	elemental sulfur, sulfite, thiosulfate	TAS [2]
MIGS-6	Habitat	marine surface sediment	TAS [2]
MIGS-15	Biotic relationship	free-living	TAS [2]
MIGS-14	Pathogenicity	none	NAS
	Biosafety level	1	NAS
	Isolation	tidal flat sediment	NAS
MIGS-4	Geographic location	Arcachon Bay, France	TAS [2]
MIGS-5	Sample collection	1996	NAS
MIGS-4.1	Latitude	44.66	NAS
MIGS-4.2	Longitude	-1.17	NAS
MIGS-4.3	Depth	surface sediment	TAS [2]
MIGS-4.4	Altitude	Sea level	TAS [2]

TAS: Traceable Author Statement (i.e., a direct report exists in the literature); NAS: Non-traceable Author Statement.

**Figure 1:**
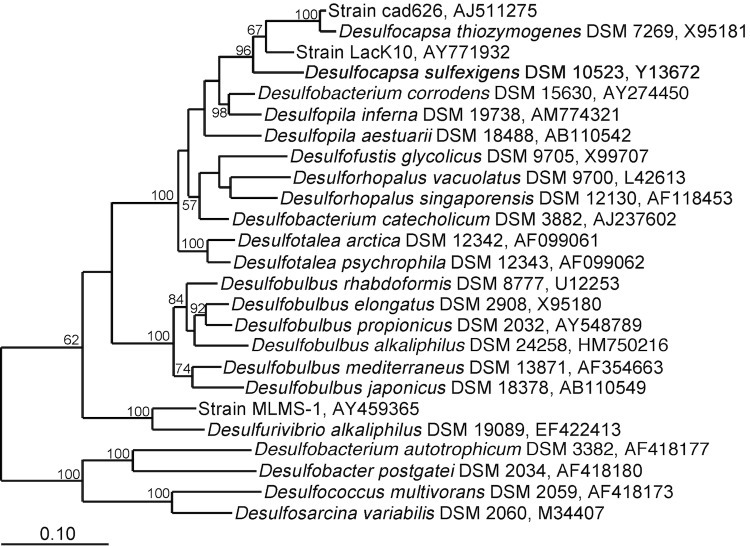
Phylogeny of *Desulfocapsa sulfexigens* based on the 16S rRNA gene. The tree was inferred from maximum likelihood analysis (RAxML [24]) with sampling of 1330 aligned sequence positions. Tree searches were performed with the general time reversible evolutionary model with a gamma-distributed rate variation across sites. Scale bar, 10% estimated sequence divergence. Values at nodes are neighbor-joining-based bootstrap percentages, calculated with Jukes Cantor distance correction and 1,000 replications.

*D. sulfexigens* SB164P1 and *D. thiozymogenes* Bra2^T^ [12] constitute the only validly published members of the genus *Desulfocapsa*, which on the basis of 16S rRNA gene sequence analysis forms a monophyletic lineage within the deltaproteobacterial family *Desulfobulbaceae* (Figure 1). So far, full genome sequences have been published for two other members of this family, *Desulfobulbus propionicus* DSM 2032 [25] and *Desulfotalea psychrophila* LSv54 [26], while genome sequences of two additional members are deposited in GenBank: *Desulfurivibrio alkaliphilus* AHT 2 (GenBank: AAQF01000000) and strain MLMS-1 (GenBank: CP001940). *D. sulfexigens* SB164P1^T^ shares less than 89% 16S rRNA gene sequence identity with any of these species (Figure 1). The lack of genome sequences of close phylogenetic relatives also adds value to the here published complete genome sequence of *D. sulfexigens*.

## Genome sequencing information

### Growth conditions and DNA isolation

The strain was grown with thiosulfate as energy source in standard bicarbonate medium at pH 7 and at 30° C [2]. Cells were harvested by centrifugation, stored at minus 80° C and shipped on dry ice to the Max Planck Institute for Molecular Genetics (Berlin, Germany). There, the DNA was isolated with the Genomic DNA kit (Qiagen, Hildesheim, Germany) according to the manufacturer's instructions, evaluated using standard procedures and sequenced.

### Genome sequencing, assembly and annotation

The genome of *D. sulfexigens* SB164P1 was sequenced using the 454 GS FLX Titanium [Table 2] pyrosequencing system (360,793 reads; Roche) combined with fosmid end-sequencing using the pCC1FOS vector (5,836 reads; Epicentre). Together, the pyrosequencing and the fosmid end-sequencing reads achieved a coverage of 32.4×. The reads were assembled in a hybrid-assembly using Newbler version 2.5.3 (Roche). Gaps in the assembly were closed using 259 reads generated by Sanger sequencing. The genome was auto-annotated using the IMG-ER pipeline [27].

**Table 2:** Genome sequencing project information

**MIGS ID**	**Characteristic**	**Details**
MIGS-28	Libraries used	2kb (pUC19) and 40kb (pcc1FOS) Sanger and 454 standard libraries
MIGS-29	Sequencing platform	ABI-3730, 454 GS FLX Titanium
MIGS-31.2	Sequencing coverage	1.1× Sanger 40kb insert, 31.3× pyrosequencing
MIGS-31	Finishing Quality	Finished
MIGS-30	Assembler	gsAssembler (Newbler) version 2.5.3
MIGS-32	Gene calling method	IMG-ER pipeline [27](CRISPR: CRT [28] and PILERCR [29]; tRNAs: tRNAScan-SE-1.23 [30]; rRNA: RNAmmer [31]; other genes: Prodigal [32])
	GenBank ID	CP003985, CP003986
	GenBank date of release	14.01.2013
	GOLD ID	Gi18068
	NCBI project ID	91121
	IMG Taxon ID	2512875001
MIGS-13	Source material identifier	DSM 10523T
	Project relevance	Sulfur cycle

### Nucleotide sequence accession numbers:

Sequences of chromosome and plasmid of *Desulfocapsa sulfexigens* have been deposited at GenBank with the accession numbers CP003985 and CP003986, respectively.

### Genome properties

In total, the genome of *D. sulfexigens* SB164P1 consists of one chromosome with a size of 3,986,761 bp (G+C content: 45% [Table 3]) and one plasmid with a size of 36,751 bp (G+C content: 44%). A total of 3,551 protein coding genes (thereof 31 on the plasmid), 46 tRNA-encoding genes, and 3 rRNA operons were predicted. Of all protein-encoding genes, 2,794 (78.7%) were auto-annotated with a functional prediction. The distribution of genes into COGs functional categories is presented in Table 4.

**Table 3:** Genome statistics.

**Attribute**	**Value**	**% of Total**
Genome size (bp)	4,023,512	
DNA coding region (bp)	3,615,930	89.87%
DNA G+C content (bp)	1,825,760	45.38%
Extrachromosomal elements (plasmids)	1	
Size of extrachromosomal element (bp)	36,751	
Total genes	3,551	100%
RNA genes	60	1.69%
rRNA operons	3	
Protein-coding genes	2,794	78.68%
Genes in paralog clusters	1,286	36.22%
Genes assigned to COGs	2,772	78.06%
Genes assigned Pfam domains	2,902	81.72%
Genes with signal peptides	764	21.52%
CRISPR count	1	

**Table 4:** Number of genes associated with the general COG functional categories

**Code**	**Genes on** **chromosome**	**Genes on** **plasmid**	**%age**	**Description**
J	175	2	5.7	Translation, ribosomal structure and biogenesis
A				
K	120	1	3.9	Transcription
L	153	8	5	Replication, recombination and repair
B	5	0	0.2	Chromatin structure and dynamics
D	33	1	1.1	Cell cycle control, cell division, chromosome partitioning
Y				
V	51	0	1.7	Defense mechanisms
T	310	0	10.2	Signal transduction mechanisms
M	250	0	8.2	Cell wall/membrane/envelope biogenesis
N	83	0	2.7	Cell motility
Z				
W				
U	93	0	3	Intracellular trafficking, secretion, and vesicular transport
O	116	0	3.8	Posttranslational modification, protein turnover, chaperones
C	256	1	8.4	Energy production and conversion
G	105	0	3.4	Carbohydrate transport and metabolism
E	216	1	7.1	Amino acid transport and metabolism
F	71	1	2.3	Nucleotide transport and metabolism
H	157	2	5.1	Coenzyme transport and metabolism
I	79	0	2.6	Lipid transport and metabolism
P	161	0	5.3	Inorganic ion transport and metabolism
Q	50	0	1.6	Secondary metabolites biosynthesis, transport and catabolism
S	230	1	7.5	Function unknown
-	859	0	-	Not in COGs

## Insights from the genome sequence

### Sulfur and energy metabolism

*D. sulfexigens* SB164P1 thrives on the disproportionation of thiosulfate, sulfite and elemental sulfur, but is unable to reduce sulfate, although it is related to sulfate reducers, of which several are able to grow by both sulfate reduction and disproportionation, e.g. *D. thiozymogenes, D. propionicus* DSM 2032 and *Desulfofustis glycolicus* DSM 9705 [2,12,33]. This is intriguing as the genome of *D. sulfexigens* SB164P1 contains the complete set of genes known to be involved in dissimilatory sulfate reduction [34] including: SulP-family sulfate permease (UWK_00097), ATP sulfurylase (UWK_02284), Mn- dependent inorganic pyrophosphatase (UWK_01588, UWK_03148), the AprA and B subunits of APS reductase (UWK_02023, UWK_02024) and the DsrA, B, C and D subunits of the dissimilatory sulfite reductase (UWK_01633, UWK_01634, UWK_01635) and DsrC (UWK_00448). Also genes encoding sulfite-reductase-associated electron transport proteins DsrPJKM (UWK_00239 – UWK_00242) are present in the genome of *D. sulfexigens* SB164P1. Thus, it is still unknown why *D. sulfexigens* SB164P1 is unable to respire sulfate.

In addition, 6 genes encoding polysulfide reductases were found (UWK_00238, UKW_02207, UWK_02291, UWK_03020, UKW_03030, UWK_03039, UWK_03284). Four of 7 polysulfide reductases form an operon with a 4Fe-4S ferredoxin iron–sulfur binding domain containing a hydrogenase and a cytochrome C family protein. They may be involved in the reduction of elemental sulfur to H_2_S [35] and are thus likely involved in hydrogenotrophic sulfur reduction - an alternative to elemental sulfur disproportionation for generating energy for *D. sulfexigens* SB164P12 [8]. The genome contains several molybdopterin oxidoreductases (UWK_01206, UWK_02209 & UWK_02642, UWK_02781) that are likely involved in sulfur metabolism either as subunits of thiosulfate or tetrathionate reductases. Thiosulfate reductase catalyzes the initial step in the disproportionation of thiosulfate, i.e. its reductive cleavage into sulfite and sulfide [8]. An operon containing genes encoding a sulfur reductase/hydrogenase beta subunit (UWK_01338), an oxidoreductase FAD/NAD(P)- binding subunit (UWK_01339), a NADH ubiquinone oxidoreductase (UWK_01340) and a sulfur reductase/hydrogenase alpha subunit (UWK_01341) was identified. Similar to the function of polysulfide reductases, this operon may encode proteins that are involved in coupling hydrogen oxidation to sulfur reduction. Finally, three genes encoding for heterodisulfide reductase subunits HdrA, HdrB and HdrC (UKW_02025, UKW_02026, UKW_02027) were found. They may be involved in the oxidation of elemental sulfur to sulfite [36], and thus replace the function of the reverse sulfite reductase in the disproportionation pathway [8], which was not found in the genome. Sulfite as an intermediate was confirmed by the observation of free sulfite in medium of cultures that grew by thiosulfate as well as by elemental sulfur disproportionation [11]. However, only genes encoding dissimilatory sulfite reductases were hitherto identified in the genome. Finally the genome encodes nine rhodanese-related sulfurtransferases that may be involved in the metabolism of thiosulfate and elemental sulfur during disproportionation (UWK_00046; UWK _ 00165; UWK _ 00611; UWK _00945; UWK _01143; UWK _01446; UWK _ 01496; UWK _03368; UWK _03369) although their specific roles in disproportionation mechanisms need to be investigated.

Inhibition experiments with the proton gradient uncoupler CCCP, the electron transport chain inhibitor HQNO [11] as well as with molybdate [2], a competitive inhibitor of sulfate reducers that interferes with the formation of activated sulfate (APS) [37], showed that *D. sulfexigens* uses both substrate level phosphorylation and the generation of proton motive force for ATP generation during disproportionation [34]. In accordance, its genome contained genes encoding a F-type ATPase. Subunits A, B and C of the F_0_ subcomplex are encoded by genes (UKW_ 00974; UWK _01665), (UWK_00972; UWK _001702; UWK _01703) and (UWK_00973; UWK _01666). The subunits α, β, γ, δ and ε of the F_1_ subcomplex are encoded by genes (UWK_00971; UWK _01705), (UWK_00978; UWK _01708), (UWK_00970; UWK _01706), (UWK_01704) and (UWK_00977; UWK _01708), respectively. The genome also encodes a proton-translocating NADH hydrogenase (UWK_03559 to UWK_03571).

### Carbon assimilation

*D. sulfexigens* SB164P1 grows autotrophically by fixing CO_2_. Accordingly, its genome encodes a complete acetyl-CoA pathway for fixing CO_2_ including the key enzymes carbon monoxide dehydrogenase catalytic subunit (UWK_03164) and acetyl-CoA decarboxylase/synthase (UWK_03163) [38]; and carbon monoxide dehydrogenase activity was observed in enzyme assay-based studies of *D. sulfexigens* SB164P1 [10]. Indirect support for an active carbon assimilation via the reversed acetyl-CoA pathway was provided by the high carbon fractionation value of 37 per mill determined by carbon isotope studies of the cell biomass [10]. Thus, *D. sulfexigens* SB164P1 appears to be able to thrive on CO_2_ as its only carbon source using a reverse acetyl-CoA pathway. This is the first report of the identity of a carbon fixation pathway of a member of the family *Desulfobulbaceae*. Notably, this pathway is shared with the sulfate reducer *Desulfobacterium autotrophicum* HRM1 of the *Desulfobacteraceae* in which it has been studied in detail [39].

Organic carbon in the form of acetate neither enhanced the growth yield nor the growth rate of *D. sulfexigens* SB164P1, indicating that CO_2_ fixation is not a growth-limiting process. Despite the fact that *D. sulfexigens* SB164P1 is unable to use organic substrates as e-donors and energy source, its genome encodes a complete TCA cycle [40]: (citrate synthase I and II (UWK_01937; UWK _00579), aconitate hydratase (UWK_01509), isocitrate dehydrogenase (UWK_01609), 2-oxo-glutarate dehydrogenase α, β, γ subunit (UWK_02894 to UWK_02896), succinyl CoA synthetase α and β subunit (UWK_01582; UWK _01584), fumarate reductase cytochrome b subunit, flavoprotein subunit and Fe-S protein subunit (UWK_03265 to UWK_03267) and malate dehydrogenase (UWK_03173). It also encodes a complete glycolysis pathway (Berg et al. 2002): Glucose-6-phosphate isomerase (UWK_01632), fructose 6-phosphate kinase (UWK_01908), fructose-1,6-bisphosphatase (UWK_03194), fructose bisphosphate aldolase (UWK_02512), triosephosphate isomerase (UWK_00786; UWK _01623), glyceraldehyde-3 phosphate dehydrogenase (UWK_01687), phosphoenol pyruvate synthase (UWK_00627; UWK__02176; UWK _02650), 3-phosphoglycerate kinase (UWK_00787), 2,3 phosphoglycerate mutase (UWK_03186) and pyruvate kinase (UWK_00304; UWK _00318; UWK _00709) are encoded in its genome. These pathways run probably in reverse in *D. sulfexigens* and are involved in the synthesis of cell material.

### Nitrogen metabolism

*D. sulfexigens* SB164P1 grows with free nitrogen gas as sole nitrogen source. Accordingly, all genes necessary for nitrogen fixation were identified in the genome [41]. They are closely linked in the genome. The derived proteins are: NifH (UWK_0033), NifHD1 and NifHD2 that function as regulator proteins (UWK_00334; UWK _00335), NifD and NifK, which constitute the α and β chain of the molybdenum-iron nitrogenase (UWK_00336; UWK _00337), a nitrogenase associated protein (UWK_00340) and NifE, NifN and NifB (UWK_00347; UWK _00348; UWK _00349). Cultures of *D. sulfexigens* reduce acetylene to ethylene in a standard nitrogen fixation assay. Thus, despite the low energy output of the sulfur disproportionation reaction *D. sulfexigens* conserves sufficient energy to grow both autotrophically and diazotrophically.

Furthermore the *D. sulfexigens* SB164P1 genome indicates a potential for dissimilatory nitrate and nitrite metabolism including an operon that contains three units of an ABC type nitrate/sulfonate/bicarbonate transport system consisting of a periplasmic (UKW_00829), a permease (UKW_00830) and an ATPase (UKW_00831) component. In addition, the genome contains two nitrate/nitrite transporters driven by electrochemical potential (UKW_02352, UKW_03309), three nitrate/TMAO reductases (UKW_02209, UKW_02550, UKW_03309), one nitrate reductase (gamma subunit) (UKW_00242), one NADPH-nitrite reductase (UKW_03259) and two hydroxylamine reductases (UKW_00765, UKW_03258). The NADPH dependent nitrite reductase is of an assimilatory type that reduces nitrite to ammonium hydroxide. Ammonium can then be assimilated by the cell. A similar set of transport systems and reductases has been reported being responsible for nitrate assimilation in *Rhodobacter capsulatus* E1F1 [42].

### Oxidative stress

The genome of *D. sulfexigens* encodes several genes involved in defense against oxidative stress such as superoxide dismutase (UWK_02392) and catalase (UWK_00321). In addition, the genome encodes the two subunits of a cytochrome *bd*-type quinol oxidase (UWK_01593; UWK _01594). This enzyme reduces oxygen with electrons from the quinone pool and may thereby protect cells from oxygen [43]. Moreover, the genome encodes 5 glutathione synthases (UWK_00572; UWK_00580; UWK _01802; UWK _03585; UWK _03624). Glutathione may serve as an antioxidant and as an oxygen scavenger [44].

As the substrates for sulfur disproportionation are mainly generated as intermediates of sulfide oxidation in the oxic-anoxic interfaces *D. sulfexigens* seems well equipped to maneuver in an environment, where it occasionally may encounter oxygen or its partly reduced intermediates. In such a habitat, the capacity to detoxify reactive oxygen species including hydroxyl- and superoxide radicals as well as hydrogen peroxide seems of key importance for survival.

### Chemotaxis and motility

The genome of *D. sulfexigens* SB 164P1 contains 10 methyl-accepting chemotaxis transmembrane proteins (UWK_00167; UWK_00267; UWK_00616; UWK_00640; UWK_00995; UWK_01396; UWK_01397; UWK_01493; UWK_01787; UWK_01890) that interact with chemotaxis signal transduction proteins CheW (UWK_00950; UWK_03012; UWK_03013). CheW is also involved in flagellar motion. In addition, we found a number of different response regulators including 32 copies of one type that was automatically annotated as a response regulator containing a CheY-like receiver AAA-type ATPase, and a DNA binding domain. This regulator receives signals from a sensor partner in a bacterial 2-component system (UKW_00056; UKW_00306; UKW_00595; UKW_00622; UKW_00625, UKW_00834; UKW_00976; UKW_01208; UKW_01271; UKW_01512; UKW_01944; UKW_01945; UKW_01945; UKW_01952; UKW_02106; UKW_02134; UKW_02287; UKW_02315; UKW_02346; UKW_02374; UKW_02508; UKW_02614; UKW_02645; UKW_02648; UKW_02788; UKW_02863; UKW_02986; UKW_03016; UKW_03064; UKW_03068; UKW_03331; UKW_03429; UKW_03516). We also found a number of other genes that are encoding parts of the chemotaxis complex such as CheB that is composed of a sensor histidine kinase and a response regulator (UKW_02813; UKW_03014), CheC that functions as a methylation inhibitor and restores the pre-stimulus level of the cell (UKW_03066; UKW_03067) and CheR, a methylase which methylates the chemotaxis receptor (UKW_03015)(see [45] for a detailed overview).

The genome contains all the genes that are required for flagellum formation [46] (FlgA, UWK_03088; FlgB, UWK_03070; FlgC, UWK_03071; FlgD, UWK_03080; FlgE, UWK_03081; FlgF, UWK_03097; FlgG, UWK_03098; FlgH, UWK_03101; FlgI, UWK_03101; FlgJ, UWK_03102; FlgK, UWK_03106; FlgL, UWK_03100; FlgM, UWK_03104; FlgP, UWK_03101; FliC, UWK_03115; FliD, UWK_03113; FliE, UWK_03072; FliG, UWK_3074; FliH, UWK_03075; FliI, UWK_03076; FliJ, UWK_03077; FlgL, UWK_03084; FliM, UWK_03085; FliN, UWK_03086; FliO, UWK_03087; FliP, UWK_03088; FliQ, UWK_03089; FliR, UWK_03090; FliS, UWK_03112; FlhA, UWK_03092; FlhB, UWK_03091; FlhF, UWK_03093). The flagellar motor consists of proteins MotA and MotB encoded by UWK_03082 and UWK_03083, respectively. A motor of this type is driven by a proton gradient. This may explain the need for ATPases, which may be used to generate a proton motive force rather than being involved in ATP synthesis.

## Conclusion

The complete genome of the marine bacterium *Desulfocapsa sulfexigens* SB164P1 provides the starting point for a detailed analysis of the pathways involved in the disproportionation of inorganic sulfur compounds including elemental sulfur, thiosulfate and sulfite. Apart from being studied in its own right sulfur disproportionation is a key process in the sulfur cycle on a global scale with significant imprints in the geological record. In addition, the increasing number of 16S rRNA gene sequences with close similarity to members of the genus *Desulfocapsa* indicates the prevalence of the process in numerous, geophysically diverse habitats.
